# Isoniazid Conjugated Magnetic Nanoparticles Loaded with Amphotericin B as a Potent Antiamoebic Agent against *Acanthamoeba castellanii*

**DOI:** 10.3390/antibiotics9050276

**Published:** 2020-05-25

**Authors:** Kawish Iqbal, Sumayah Abdelnasir Osman Abdalla, Ayaz Anwar, Kanwal Muhammad Iqbal, Muhammad Raza Shah, Areeba Anwar, Ruqaiyyah Siddiqui, Naveed Ahmed Khan

**Affiliations:** 1 HEJ Research Institute of Chemistry, International Center for Chemical and Biological Sciences, University of Karachi, Karachi 75270, Pakistan; kawishiqbal02@gmail.com (K.I.); kanwal@hotmail.com (K.M.I.); raza_shahm@yahoo.com (M.R.S.); 2 Department of Biological Sciences, School of Science and Technology, Sunway University, Subang Jaya 47500, Selangor, Malaysia; bitabdelnasir@gmail.com (S.A.O.A.); areeba_anwar@ymail.com (A.A.); 3 Department of Biology, Chemistry and Environmental Sciences, College of Arts and Sciences, American University of Sharjah, Sharjah 26666, UAE; rsiddiqui@aus.edu

**Keywords:** magnetic nanoparticles, isoniazid, amphotericin B, pathogenic free-living amoebae, *Acanthamoeba castellanii*

## Abstract

The pathogenic free-living amoeba, *Acanthamoeba castellanii*, is responsible for a rare but deadly central nervous system infection, granulomatous amoebic encephalitis and a blinding eye disease called *Acanthamoeba* keratitis. Currently, a combination of biguanides, amidine, azoles and antibiotics are used to manage these infections; however, the host cell cytotoxicity of these drugs remains a challenge. Furthermore, *Acanthamoeba* species are capable of transforming to the cyst form to resist chemotherapy. Herein, we have developed a nano drug delivery system based on iron oxide nanoparticles conjugated with isoniazid, which were further loaded with amphotericin B (ISO-NPs-AMP) to cause potent antiamoebic effects against *Acanthamoeba castellanii*. The IC_50_ of isoniazid conjugated with magnetic nanoparticles and loaded with amphotericin B was found to be 45 μg/mL against *Acanthamoeba castellanii* trophozoites and 50 μg/mL against cysts. The results obtained in this study have promising implications in drug discovery as these nanomaterials exhibited high trophicidal and cysticidal effects, as well as limited cytotoxicity against rat and human cells.

## 1. Introduction

Free-living amoebae *Naegleria fowleri*, *Balamuthia mandrillaris*, and *Acanthamoeba castellanii* cause the central nervous system infections, primary amoebic meningoencephalitis (PAM) and granulomatous amoebic encephalitis (GAE) [1]. These diseases are rare but almost always prove to be fatal with a staggering mortality rate of over 95% [2]. Currently, there is no single effective therapeutic drug and these diseases are managed by a mixture of drugs including amphotericin B, miltefosine, voriconazole, etc. [3]. Despite rigorous chemotherapy with these drugs, the success rate is negligible. Therefore, there is an urgent need to develop an efficient drug regimen that is safer and more potent.

Nanoparticles (NPs) have attracted recent scientific attention as antimicrobial agents against a variety of microorganisms including fungi, viruses, bacteria and parasites [4,5]. Their smaller size and large surface to volume ratio makes them ideal candidates for drug delivery applications [6]. Nanoparticles, including silver, gold and iron oxide nanoparticles, are widely utilized as antibacterial agents, due to their possible interaction with DNA and inherent property of generating reactive oxygen species [7,8]. Among metallic NPs, metallic oxide nanoparticles including ZnO, CuO, Fe_2_O_3_, NiO, MgO, etc. have shown potential as antibacterial agents due to their involvement in several mechanisms, including bacteriostatic effects [9], reactive oxygen species (ROS) induction [10], and surface functionalization with peptides, antibodies and other therapeutic agents [11]. However, current utilities of nanomaterials against pathogenic free-living amoebae have limited therapeutic implications as their mode of action and in vivo potential have not been studied extensively [12,13,14,15,16].

Among nanoparticles, the most widely used metals include gold, silver and titanium oxide. Iron plays a key role in the living body. About 70 percent of iron is found in hemoglobin in red blood cells and in myoglobin in muscular cells, and about 6% of iron chelates with proteins and helps in metabolic processes. Iron based compounds have immense medicinal values and are proactively utilized as markers for the treatment of a variety of diseases including Alzheimer’s, malaria, and Parkinson’s disease [17,18,19]. Iron oxide nanoparticles gained significant importance in the field of biomedicine because of their biocompatibility, low hemolytic effect and response against external stimuli such as magnetic fields, temperature and pH [20,21,22,23]. Various studies have shown the antibacterial characteristics of iron oxide nanoparticles against a variety of bacterial strains [24,25]. Several surface engineered iron oxide nanoparticles enhance the bactericidal potential of various therapeutic agents [26,27]. Iron oxide nanoparticles are also utilized as MRI agents and in magnetic hyperthermia [28,29]. Iron oxide NPs are capable of generating reactive oxygen species in the presence of UV radiation. Furthermore, conjugation with broad spectrum multiple antibiotics can be an effective strategy against *A. castellanii* but this area of research remains unexplored.

Isoniazid (isonicotinylhydrazide) is an antibiotic that is effective against tuberculosis [30]. Isoniazid inhibits the mycobacterial cell wall after activation by catalase-peroxidase enzyme [31]. It is also known to block another enzyme; fatty acid synthase [32]. In this process, it also produces nitric oxide free radicals and inhibits cytochrome P450 [33]. These enzymes are also known to play a pivotal role in pathogenic free-living amoebae. On the other hand, amphotericin B has been used in combination with other drugs against amoebae [34,35,36].

Herein, we report for the first time the antiamoebic effects of amphotericin B loaded on surface functionalized isoniazid-coated iron oxide nanoparticles against *A. castellanii* belonging to the T4 genotype. We used amphotericin B as it shows limited effects against *Acanthamoeba* and the purpose of this study was to determine whether such a formulation can enhance its effects. The novel bionanomaterial was synthesized by coupling of modified isoniazid over magnetic nanoparticles. These nanoparticles were thoroughly characterized by nuclear magnetic resonance spectroscopy, Fourier transformation infrared spectroscopy, dynamic light scattering, and atomic force microscopy. They were also tested for their application against *A. castellanii*. These bionanomaterials were subjected to cytotoxicity against 3T3 fibroblasts and HeLa adenocarcinoma cells in vitro and hemocompatibility assays prior to study of their antiamoebic effects. Finally, these nanoparticles were tested against both trophozoite and cyst stages of *A. castellanii* and were found to possess potent antiamoebic activity.

## 2. Methods and Materials

### 2.1. Chemicals

All organic solvents utilized in experiments were of HPLC grade. Ferric sulphate hexahydrate Fe_2_(SO_4_)_3_.6H_2_O, ferrous sulphate heptahydrate (FeSO_4_.6H_2_O), 3-(trimethoxysilyl)propyl methacrylate (MPTES), 4-dimethyl amino pyridine (DMAP), azobisisobutyronitrile (AIBN), ammonium hydroxide, dicyclohexylcarbodimide (DCC), isoniazid and amphotericin B were obtained from Sigma-Aldrich (San Francisco, CA, USA) and Merck (Darmstadt, Germany).

### 2.2. Synthesis of N′-Methacryloylisonicotinohydrazide (MIH)

**Figure 1:**
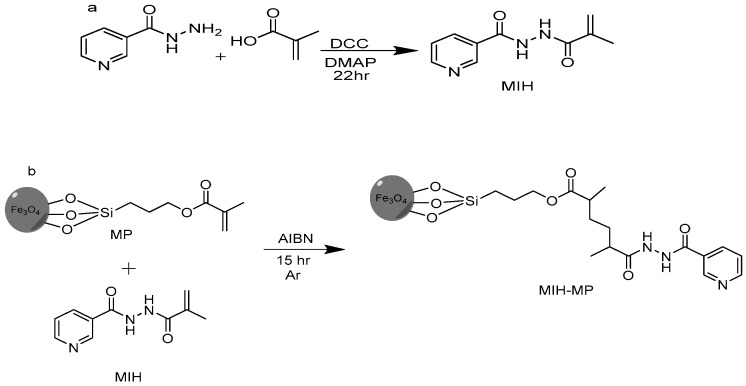
(**a**) Synthetic scheme of MIH molecule. (**b**) MIH-MP nanoparticles.

Methacrylic acid (1.29 g, 15.0 mmol), 4-dimethyl amino pyridine (0.061 g, 0.5 mmol) and dicyclohexylcarbodimide (DCC, 3.10 g, 15.0 mmol) were taken in a round bottom flask containing THF (50 mL) connected with a condenser. The reaction was stirred for 10 min at 60 °C under an argon atmosphere. Isoniazid (0.68 g, 7.29 mmol) was added later and the reaction was refluxed for 22 h followed by its monitoring via thin layer chromatography using DCM and MeOH (9:1 *v*/*v*) as a mobile phase (Figure 1a). The resulting mixture was concentrated in vacuo and the concentrate was then subjected to column chromatography using flash silica as a stationary phase. The desired compound was obtained using hexane and ethyl acetate (6:4 v/v) as a mobile phase. Rf: 0.56 (DCM:MeOH, 9:1, *v*/*v*), yield 50%, melting point (M.P.): 160–170 °C. EI-MS: m/z 205.1, ^1^H NMR (300 MHz MeOD) δ ppm: 8.701 (d 2H pyridine), 7.853 (d 2H pyridine), 5.866 (s 1H C=C), 5.514 (s 1H C=C), 3.02 (s 3H CH_3_).

### 2.3. Functionalization of Magnetic Nanoparticles (MPs) with MIH

Firstly, iron oxide magnetic nanoparticles of narrow size distribution were prepared by precipitating Fe (III) and Fe (II) in alkaline medium by the co-precipitation method [37]. Then, synthesized MNPs were surface functionalized with 3-(trimethoxysilyl)propyl methacrylate (MPTES) in a ratio of 1:6 by silanization reaction [38]. MIH conjugation on methacrylate-coated MNPs was achieved through raft polymerization method. Briefly, to a solution of MPTES-modified MNPs (0.15 g) in 30 mL anhydrous acetonitrile (ACN) under an argon atmosphere, isonicotinohydrazide (0.950 g, 4.63 mmol) was added after being stirred for 10 min at 60 °C. Azobisisobutyronitrile (AIBN) (1.3 g, 7.91 mmol) was then added to the resulting mixture and was refluxed for 15 h under an argon atmosphere at 60 °C (Figure 1b). The resulting nanoparticles (MIH-MPs) were washed sequentially with ACN to remove impurities, followed by magnetic decantation and drying at 60 °C to obtain product.

### 2.4. Characterization: Size, Size Distribution and Morphology

The average hydrodynamic diameter and polydispersity index (PDI) of the nanopartciles were investigated via Zetasizer (Zetasizer Nano ZS90 Malvern Instruments, Malvern, UK). Concisely, diluted nanoparticles were transferred to a transparent plastic cuvette with caution to avoid any bubble formation. The cuvette was then placed in the cell holder of the instrument and analysis was taken at 90 degree scattering at 25 °C. The medium viscosity and refractive index were constant and kept at 1.0, 1.33 and 80.4 mPa respectively. Nanoparticles were also characterized for morphology using atomic force microscopy (AFM, Agilent 5500). A drop of formulation was placed on a mica slide, air dried at ambient temperature and then placed under a microscope. The morphology was investigated in the non-contact mode.

### 2.5. Drug Loading Studies

The drug entrapment via adsorption was achieved by incubating amphotericin B (Amp B) along with synthesized MIH-MP. Briefly, both Amp B and nanoparticles were mixed together in water at various equivalents in methanol and incubated for 24 h at room temperature in a rotary shaker at 200 rpm. Drug-loaded nanoparticles were removed from the liquid by means of a permanent magnet and stored at 4 °C for further analysis.

### 2.6. Drug Entrapment Efficiency Determination

The drug-loaded MIH-MP nanoparticles were decanted via magnetization and the supernatant containing unloaded drug was diluted 100 times in water. The diluted supernatant was then quantified at 405 nm by UV spectroscopy (Shimadzu 1800 series, Shimadzu, Kyoto, Japan). Drug entrapment was investigated by the following relation.

**%EE = (Q_t_ − Q_p_)/Q_t_ × 100**

**Q_p_**: Quantity of free drug.
**Q_t_**: Quantity of drug added.
**%EE**: Entrapment efficiency of loaded drug in percent.

#### FTIR Spectroscopy

Fourier transformed infrared (FT-IR, IR-470 spectrometer (Shimadzu, Kyoto, Japan)) analysis was performed in order to elucidate the possible drug entrapment and surface functionalization. The powdered nanoparticles (2 mg) were mixed with KBr and subjected to high pressure of 200 Psi to obtain self-supporting disks.

### 2.7. Hemocompatibility Study

Human blood was obtained from healthy individuals in the University of Karachi, Pakistan, following the relevant guidelines and regulations. EDTA-stabilized fresh human blood samples (5.0 mL) were added to 10 mL of PBS. Then, red blood cells (RBCs) were isolated via centrifugation at 6000 rpm and washed several times with PBS solution. The purified RBCs were further diluted in 50 mL PBS and Triton X-100 was used as the positive control respectively. Then, 0.2 mL of diluted RBC suspension and 0.8 mL of MIH-MP solutions in a range of 200–1000 µg/mL were mixed via vortexing. All sample tubes were kept in a static condition at room temperature for 3 h. Finally, the mixtures were centrifuged at 12,000 rpm for 10 min, and 1.5 mL of supernatant of each sample was transferred to a cuvette. The absorbance values of the supernatants at 540 nm were determined by UV-vis spectrophotometer. The percent hemolytic activity of RBCs was calculated using the following relation.

**% H.A = Rs/Rc × 100**

**Rs**: Absorbance of sample
**Rc**: Absorbance of positive control
**% H.A**: Hemolytic activity in percent

### 2.8. In Vitro Cytotoxicity

The synthesized nanocarriers were screened for cytotoxicity using the MTT assay. Both 3T3/NIH and HeLa cells were grown in Dulbecco’s modified Eagle’s medium (DMEM) with fetal bovine serum (10%) and antibiotics (streptomycin and penicillin, about 50 U/mL) at 37 °C and under carbon dioxide (5%) humid atmosphere. Both cell lines were incubated on 96-well plates at 8 × 10^3^ and 6 × 10^4^ cells/well in 200 µL of refined media. After approximately 24 h incubation, fresh media was introduced (200 µL) with NPs at various concentrations up to 100 μg/mL. Incubated cells in media without NPs were used as negative control and the treatment time was 48 h. 3-(4,5-Dimethylthiazol-2-Yl)-2,5-diphenyltetrazolium bromide solution (MTT) in PBS was introduced in each well (20 µL; 5 mg/mL). TheMTT reagent was removed after 4 h incubation. The resulting formazan crystals were dissolved and introduced into 200 μL DMSO per well and analyzed at 570 nm in a microplate reader. For positive control and reference standard, doxorubicin and cyclohaxanamide were used. The following relation was utilized to calculate the % cell viability.

**Cell viability % = At/Ac × 100** (1)

**At**: Mean of Absorbance value of Test Sample.
**Ac**: Mean of Absorbance value of Control.

### 2.9. Acanthamoeba Culture

*A. castellanii* was cultured in growth medium (PYG) consisting of Proteose peptone (0.75% *w*/*v*), yeast extract (0.75% *w*/*v*), and D-glucose (1.5% *w*/*v*) [39].

### 2.10. Antiamoebic Assay

Antiamoebic assays were conducted by the procedure reported previously [40]. Briefly, amoebae were seeded in 24 well plates at 5 × 10^5^/well/500 µL RPMI. Later, cells were incubated with different concentrations of nanoparticles. Chlorhexidine was used as a positive control against *A. castellanii*. Amoebae alone were used as a negative control, while the solvent used was also added as additional solvent control. After 24 h incubation at 30 °C with the compounds, the trophozoites were enumerated by trypan blue (0.1%) exclusion assay, using a haemocytometer.

### 2.11. Anticystic Assay

*A. castellanii* cysts were prepared by inoculating the amoeba trophozoites on non-nutrient agar plates and incubating at 30 °C for 14 days. Later, cysts were scraped using PBS with the help of a cell scraper, centrifuged at 1260× *g* for 10 min and resuspended in 10 mL PBS. For the cysticidal experiment against *A. castellanii*, cysts (1 × 10^5^/well/500 µL PYG) were incubated with nanoconjugates at 30 °C for 72 h. After the incubation time, the remaining viable cysts were counted by the trypan blue exclusion method, using a hemocytometer as described previously [40].

## 3. Results and Discussion

### 3.1. Synthesis of N′-Methacryloylisonicotinohydrazide (MIH)

The ^1^H NMR spectra of synthesized MIH compound showed two aromatic doublets around δ 7.8 ppm and 8.7 ppm of 2H. Two olefinic protons appeared as a singlet around δ 5.5 ppm and 5.8 ppm. A singlet was also observed around δ 3.02 ppm, which corresponds to a methyl group. The EI-MS spectrum of synthesized MIH showed M^+^ at m/z 205, which was the theoretical weight of the compound with the formula C_10_H_11_N_3_O_2_.

### 3.2. Preparation of MIH-Coated MNPs

The synthesized magnetite nanoparticles showed characteristic absorption around 630 cm^−1^, which was attributed to vibration of Fe–O bonds. Furthermore, absorptions at 1614 cm^−1^ and 3413 cm^−1^ corresponded to stretching and bending vibrations of hydroxyl groups on the surface of the nanoparticles [41]. The induction of MPTES induced characteristic absorption around 1643 cm^−1^ and 1772 cm^−1^, corresponding to an α, β unsaturated bond and ester (C=O) respectively [42]. A frequency of 1050 cm^−1^ was observed in the fingerprint region corresponding to C–O bending. Fabrication of MIH on silane-coated nanoparticles gave a pair of frequencies at 1744 cm^−1^ and 1689 cm^−1^, corresponding to the C=O of an ester and amide. A stretch around 1465–1562 cm^−1^ corresponded to an aromatic ring, and stretching and bending vibration around 3456 cm^−1^ and 1641 cm^−1^ corresponded to N–H. The characteristic absorption was evidence for successive functionalization of the MIH molecule on the surface of the nanoparticles.

### 3.3. Size, Size Distribution and Morphology

**Figure 2:**
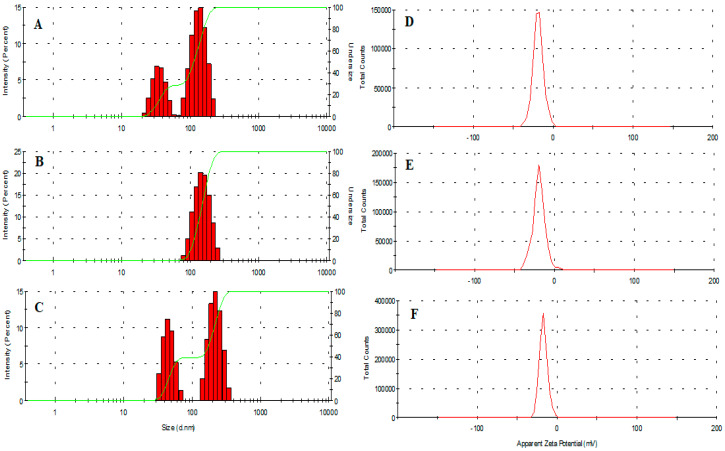
(**A**) Average hydrodynamic diameter of synthesized MIH-MP. (**B**) MIH-MP-AMP nanoparticles at 1:1 AMP and nanoparticles. (**C**) MIH-MP-AMP nanoparticles at 2:1 ratio AMP to nanoparticles. (**D**) Zeta potential of developed MIH-MP. (**E**) Zeta potential of MIH-MP-AMP at 1:1 AMP and nanoparticles. (**F**) Zeta potential of MIH-MP-AMP at 2:1 AMP to nanoparticles.

The average hydrodynamic diameter of vacant and amphotericin B-loaded MIH-MP nanoparticles is presented in Table 1. AmpB-loaded MIH-MP nanoparticles are comparatively larger in size (184 ± 2.7 nm) at 1:1 in comparison with unentrapped nanoparticles (140.2 (62% population)), which shows the successive stacking of AmpB onto the surface of nanoparticles (Figure 2A–C). Interestingly drug entrapped nanoparticles are found to have more negative zeta potential (Table 1), which depicts the colloidal stability of nanoformulation (Figure 2D–F). Particle agglomeration is less likely to occur in nanoparticles with a high surface potential due to higher repulsion potential through which they attain long-term stability. The values indicate the successive adsorption of drug on nanoparticles; Table 1 shows the percentages for each population from DLS.

**Figure 3:**
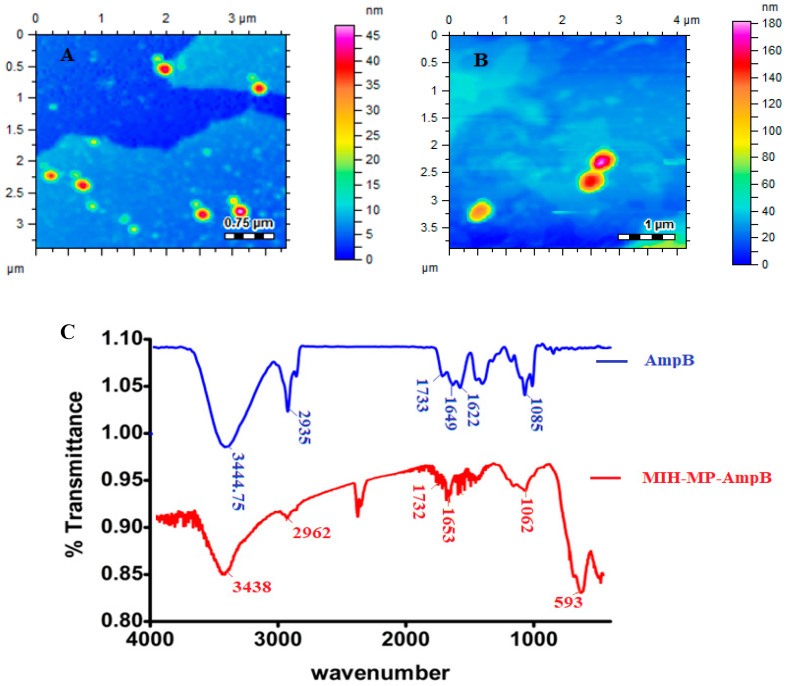
(**A**) Morphology of vacant MIH-MP nanoparticles. (**B**) Morphology of MIH-MP-AmpB nanoparticles. (**C**) Fourier transformed infrared spectra of Amp B along with MIH-MP-AmpB.

The polydispersity index (PDI) describes the uniform dispersion of a colloidal suspension. A PDI more than 0.3 indicates the broad size distribution of the particles [43]. In the current study, the PDI of empty and AmpB-loaded MIH-MP nanoparticles is found to have uniform distribution as represented in Table 1. The vacant MIH-MP nanoparticles were nearly spherical in morphology as revealed by AFM (Figure 3A). However, the amphotericin B-loaded MIH-MP nanoparticles showed a slight distortion in morphology (Figure 3B), which may be evidence of successive entrapment of the drug on the nanoparticles.

### 3.4. Drug Hosting Studies

MIH-MP nanoparticles entraped 76.30 ± 1.34% of drug at a 1:1 ratio, which is significantly higher as compared to the 2:1 ratio (64.34 ± 4.55%) (Table 1). The higher drug entrapment efficiency may be attributed to π−π stacking and hydrogen bonding interactions between drug molecules and carrier. Therefore, the 1:1 formulation was used in further studies.

### 3.5. FTIR Spectroscopy

Fourier transformed infrared analysis was conducted in order to identify the possible stacking of the drug in nanoparticles (Figure 3C). Amphotericin B revealed characteristic absorption around 1733 cm^−1^, 1622 cm^−1^ and 1649 cm^−1^, corresponding to C=O, NH bending and C=C moieties [44]. The stretching frequency at 3444.75 cm^−1^ corresponds to OH stretching. AmpB-loaded MIH-MP nanoparticles show slight variation in absorption frequencies, where the peak at 1733 cm^−1^ of carboxylic acid (C=O) was shifted to 1732 cm^−1^ and the peak at 1649 cm^−1^ was shifted to 1653 cm^−1^. The frequency at 1085 cm^−1^ of the acetal bond was shifted to 1062 cm^−1^, which may be attributed to increased secondary hydrogen bonding and the π−π stacking interaction in between the drug and the synthesized NPs.

### 3.6. Hemocompatibility

**Figure 4:**
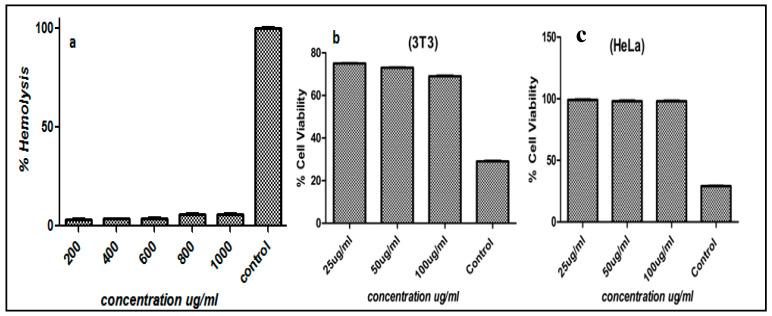
(**a**) Percent hemolysis of MIH-MP nanoparticles at different concentrations. (**b**) In vitro cytotoxicity profile of MIH-MP nanoparticles against 3T3(NIH) and (**c**) HeLa cell lines.

The interaction of surface functionalized magnetic nanoparticles with negatively charged membranes has been studied via a hemolysis study [45]. The release of hemoglobin from cells determines the membrane destruction characteristics of nanoparticles. Titron X-100 was used as 100% values for erythrocytes, respectively. MIH-MP nanoparticles were taken at different concentration ranges from 200, 400, 600, 800 and 1000 µg/mL, and released hemoglobin was quantitatively analyzed at 541 nm (Figure 4a). The nanoparticles at higher concentrations showed less than 10% hemolytic activity, showing the membrane friendly properties (i.e., did not cause any disruption to the biological membrane) [46] of synthesized MIH-MP nanoparticles.

### 3.7. In Vitro Cytotoxicity

Mouse embryonic fibroblast 3T3 and human cervical HeLa cell lines are commonly used cell lines that show reproducibility. MIH-MP nanoparticles were exposed to 3T3 and HeLa cell lines for the evaluation of cytotoxicity using the MTT assay. For comparison, doxorubicin and cyclohexanamide were used as positive controls. MIH-MP nanoparticles were incubated at various concentrations against 3T3 and HeLa cell lines. MIH-MP nanoparticles showed cell viability in a concentration dependent manner. Experiments conducted on 3T3 cell lines showed cell viability of about 70 ± 1.2% after 48 h. Similarly, cell viability against HeLa cell lines was found to be 98 ± 0.2% at a concentration of 100 μg/mL, as depicted in Figure 4b,c. The results revealed that the synthesized nanoparticles are biocompatible, which is also supported by other studies where magnetic nanoparticles are shown to cause minimal toxicity in living organisms [47].

### 3.8. Isoniazid-Nanoparticles-Amphotericin B Displayed an IC_50_ of 45 μg/mL against A. castellanii Trophozoites

**Figure 5:**
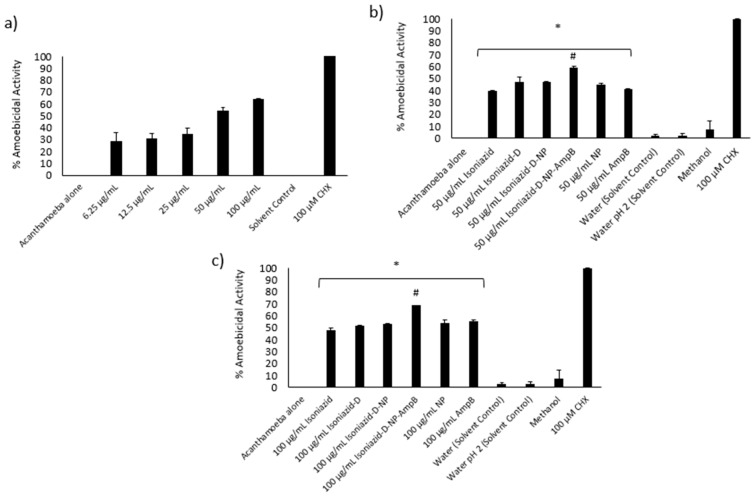
(**a**) Determination of MIC of MIH-MP-Amp B against *A. castellanii* trophozoites. (**b**) Amoebicidal assay against *A. castellanii* trophozoites at 50 µg/mL. (**c**) Amoebicidal *A. castellanii* trophozoites at 100 µg/mL. (* *p* < 0.05 as compared to negative control, # *p* < 0.05 as compared to drugs and nanoparticles alone).

Amoebicidal assays were conducted to determine the effects of various concentrations of isoniazid-np-amphotericin B on the viability of *A. castellanii*. Treatment with isoniazid-np-amphotericin B was observed to inhibit the viability of amoebae, and the IC_50_ was determined to be 45 μg/mL (Figure 5a). As the IC_50_ of isoniazid-amphotericin B nanoparticles against cysts was found to be 50μg/mL (described later), subsequent assays were performed using 50 and 100 μg/mL of isoniazid-np-amphotericin B to achieve an IC_50_ and total kill. The controls used were 50 and 100 μg/mL of isoniazid alone, isoniazid derivative alone, isoniazid derivative conjugated iron oxide nanoparticles, iron oxide nanoparticles alone, and AmpB alone. Untreated amoebae were taken as the negative control and treatment of amoebae with 100 μM chlorhexidine was regarded as the positive control. When treated with 50 μg/mL isoniazid-np-amphotericin B, a 59% inhibition of *A. castellanii* viability was seen (Figure 5b). This finding was statistically significant compared to isoniazid alone, ampB alone and nanoparticles alone. Increasing the concentration to 100 μg/mL, isoniazid-np-amphotericin B showed a significant increase in inhibition compared to 100 μg/mL isoniazid-np, isoniazid alone, and amp B alone (Figure 5c). The solvents used (water and methanol) did not exhibit any inhibition of viability of *A. castellanii*.

### 3.9. Isoniazid-Nanoparticles-Amphotericin B Displayed an IC_50_ of 50 μg/mL against A. castellanii Cysts

**Figure 6:**
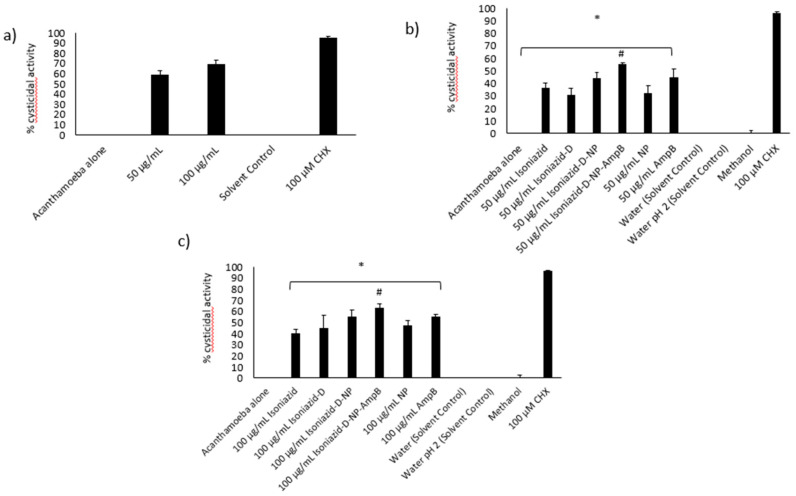
(**a**) Determination of the MIC of MIH-MP-Amp B against *A. castellanii* cysts. (**b**) Amoebicidal assay against *A. castellanii* cysts at 50 µg/mL. (**c**) Amoebicidal *A. castellanii* cysts at 100 µg/mL. (* *p* < 0.05 as compared to negative control, # *p* < 0.05 as compared to drugs and nanoparticles alone).

To assess the effects of treatment with isoniazid-np-amphotericin B on the development of cysts of *A. castellanii* into trophozoites, various concentrations of the compound were utilized in cysticidal assays. Inhibition of excystation of *A. castellanii* cysts was observed and the IC_50_ was calculated to be 50 μg/mL (Figure 6a). Subsequent assays were performed using 50 and 100 μg/mL of isoniazid-nanoparticles-amphotericin B. The negative control used was untreated cysts in growth medium (PYG) and treatment with 100 μM chlorhexidine was used as the positive control. Treatment of *A. castellanii* cysts with 50 μg/mL isoniazid-nanoparticles-amphotericin B was noticed to inhibit the excystment of cysts significantly (55%) in comparison to the negative control (Figure 6b). Treatment with a higher concentration (100 μg/mL) of isoniazid-nanoparticles-amphotericin B resulted in a 63% cysticidal activity (Figure 6c).

Tropical diseases caused by protist pathogens are still considered as neglected diseases and progress in their drug development has faced various bottlenecks [48]. Among the rare but deadly parasites, pathogenic free-living amoebae are of immense importance due to the lack of availability of any effective drugs and a high mortality rate [49]. Pathogenic free-living amoebae including *Acanthamoeba castellanii*, *Balamuthia mandrillaris* and *Naegleria fowleri* have the ability to infest and damage the brain of their hosts. Despite the failure in the development of a successful chemotherapeutic regimen, currently available drugs have limited efficacy against pathogenic free-living amoebae due to their ability to convert from trophozoites to cysts, which are highly resistant [50].

The most significant property of any nanocarrier is its ability to carry the drug cargo with it, which depicts the overall success of the therapy. High drug entrapment efficiency is essential in order to deliver therapeutic substances at the desired site of action in a significant amount, thus resulting in better therapeutic efficacy. Nanoparticulate frameworks that entrap significant amounts of drug are able to maintain controlled release behavior at a targeted site and minimize the risk of over dosage and its linked toxicities [51,52]. The most predominant factor for any drug delivery excipient is its biocompatibility, and, prior to administration in vivo, cytotoxicity is considered an essential factor [51]. Magnetic nanoparticles have been widely used as drug delivery systems for antibiotics and natural products. Lajevardi et al. (2018) reported a porous nanocomposite cephalexin delivery system based on iron oxide and silica nanoparticles [53]. In another report, citric-acid/α-cyclodextrin-functionalized Fe_3_O_4_ nanoparticles have been used as a nanocarrier for the flavonoid quercetin [54]. These reports demonstrate the efficient drug delivery ability of magnetic nanoparticles. Furthermore, metallic nanoparticles and niosomal drug delivery applications are also commonly found in the literature. Recently, a cephalexin-loaded niosomal formulation based on span 60 and tween 60 exhibited promising antibacterial effects against multi-drug resistant bacteria [55]. More recently, doxycycline-loaded niosomes showed enhanced antibacterial and anticancer potential [56]. In this study, experiments revealed that MNPs were biocompatible at less than 100 μg/mL. Surface functionalization with biocompatible molecules not only lowered toxicity but also enabled them to carry drug cargo; however, their exact mode of action needs further study.

## 4. Conclusions

In conclusion, isoniazid-conjugated iron oxide nanoparticles loaded with amphotericin B were synthesized by chemical and physical interaction. These nanoparticles were thoroughly characterized by FT-IR, AFM, and DLS analyses. They were found to be biocompatible when tested against human and rat cell lines in vitro. These nanoparticles were designed to exhibit potent antiamoebic effects against the pathogenic amoeba *A. castellanii*. The results from antiamoebic studies against *A. castellanii* showed that these nanoparticles exhibited potent amoebicidal and cysticidal effects. Furthermore, the magnetic nature of these nanoparticles can be utilized to physically isolate the parasites from biological systems and water samples, which can be of potential interest in biomedical applications. Hence, these nanoparticles are novel antiamoebic agents that should be further studied for mode of action and animal studies for the development of efficient therapeutics against infections caused by *A. castellanii*.

## Figures and Tables

**Table 1:** Average size distribution, zeta potential, PDI, drug entrapment efficiency at several ratios of MIH-MP nanoparticles. The percentages for each population from DLS is shown. Since the 1:1 formulation provided optimal drug loading, only this was used for further studies.

Nanoparticles	Ratios (Durg:NPs)	Size (nm)	PDI	Zeta Potential (mV)	Entrapment Efficiency (%)
**MIH-MP**	N. A.	140.2 ± 0.45 (62%)	0.237 ± 0.019	−17.7 ± 0.40	N. A.
**MIH-MP-AmpB**	1:1	184 ± 2.7	0.265 ± 0.04	−20.2 ± 0.41	76.30 ± 1.34
**MIH-MP-AmpB**	2:1	186.94 ± 1.20 (71%)	0.346 ± 0.043	−18.3 ± 0.92	64.34 ± 4.55

## Data Availability

Data will be provided upon request on a case-by-case basis.
